# Retraction: The Msx1 Homeoprotein Recruits G9a Methyltransferase to Repressed Target Genes in Myoblast Cells

**DOI:** 10.1371/journal.pone.0288411

**Published:** 2023-07-05

**Authors:** The PLOS ONE Editors

Following the publication of this article [1], concerns were raised regarding results presented in Figs 1 and 4. Specifically,

- The following panels appear similar:
  ○ Fig 1C C2C12 cells Msx1 panel of [1] and Fig 4D C2C12 cells Msx1 panel of [2].
  ○ Fig 1C Limb Msx1 panel of [1] and Fig 4F Limb Msx1 panel of [2].
  ○ Fig 1D Msx1 Input panel of [1] and Fig 4E Msx1 Input panel of [2].
  ○ Fig 4B Actin panel of [1] and Fig 5B Actin panel of [2] despite these results representing different experimental conditions.
  ○ Fig 4B Tubulin panel of [1] and Fig 5B Tubulin panel of [2] despite these results representing different experimental conditions.
  ○ Fig 4C H3(total) panel of [1] and Fig 5B H3(total) panel of [2] despite these results representing different experimental conditions.
- There appear to be vertical discontinuities suggestive of image splicing in the following panels in Fig 4 in [1]:
  ○ Fig 4B G9a panel, between lanes 1 and 2
  ○ Fig 4C H3K27me3 panel, between lanes 1 and 2
  ○ Fig 4C H3K9me2 panel, between lanes 1 and 2
  ○ Fig 4E Vector MHC panel, between lanes 1 and 2
  ○ Fig 4E Msx1 MHC panel, between lanes 1 and 2
  ○ Fig 4E Msx1 Actin panel, between lanes 1 and 2

The corresponding author stated that the results presented in [1] and [2] were obtained during experiments that were carried out in parallel, and indicated that the similar panels represent results obtained from the same experiments. However, this does not resolve the concern pertaining to the similar panels that have been used to represent different experimental conditions. The corresponding author stated that they were unable to comment on the image splicing concerns and the concerns pertaining to the similar panels that have been used to represent different experimental conditions as the original underlying data are no longer available.

In the absence of the original underlying data, the journal is unable to fully resolve the above concerns.

In light of the concerns affecting multiple panels in Fig 4 that question the integrity of these data, the *PLOS ONE* Editors retract this article.

CAS agreed with the retraction. JW either did not respond directly or could not be reached.
